# Radiomics-based model for predicting early recurrence of intrahepatic mass-forming cholangiocarcinoma after curative tumor resection

**DOI:** 10.1038/s41598-021-97796-1

**Published:** 2021-09-15

**Authors:** Yong Zhu, Yingfan Mao, Jun Chen, Yudong Qiu, Yue Guan, Zhongqiu Wang, Jian He

**Affiliations:** 1 Department of Radiology, Jiangsu Province Hospital of Chinese Medicine, The Affiliated Hospital of Nanjing University of Chinese Medicine, Nanjing, 210097 Jiangsu Province China; 2 Department of Radiology, Nanjing Drum Tower Hospital, The Affiliated Hospital of Nanjing University Medical School, No. 321 Zhongshan Road, Nanjing, 210008 Jiangsu Province China; 3 Department of Pathology, Nanjing Drum Tower Hospital, The Affiliated Hospital of Nanjing University Medical School, No. 321 Zhongshan Road, Nanjing, 210008 Jiangsu Province China; 4 Department of Hepatopancreatobiliary Surgery, Nanjing Drum Tower Hospital, The Affiliated Hospital of Nanjing University Medical School, No. 321 Zhongshan Road, Nanjing, 210008 Jiangsu Province China; 5 School of Biomedical Engineering, Shanghai Jiao Tong University, No.1954 Huashan Road, Shanghai, 200000 China

**Keywords:** Cancer, Medical research, Oncology, Risk factors

## Abstract

To investigate the ability of CT-based radiomics signature for pre-and postoperatively predicting the early recurrence of intrahepatic mass-forming cholangiocarcinoma (IMCC) and develop radiomics-based prediction models. Institutional review board approved this study. Clinicopathological characteristics, contrast-enhanced CT images, and radiomics features of 125 IMCC patients (35 with early recurrence and 90 with non-early recurrence) were retrospectively reviewed. In the training set of 92 patients, preoperative model, pathological model, and combined model were developed by multivariate logistic regression analysis to predict the early recurrence (≤ 6 months) of IMCC, and the prediction performance of different models were compared using the Delong test. The developed models were validated by assessing their prediction performance in test set of 33 patients. Multivariate logistic regression analysis identified solitary, differentiation, energy- arterial phase (AP), inertia-AP, and percentile50th-portal venous phase (PV) to construct combined model for predicting early recurrence of IMCC [the area under the curve (AUC) = 0.917; 95% CI 0.840–0.965]. While the AUC of pathological model and preoperative model were 0.741 (95% CI 0.637–0.828) and 0.844 (95% CI 0.751–0.912), respectively. The AUC of the combined model was significantly higher than that of the preoperative model (*p* = 0.049) or pathological model (*p* = 0.002) in training set. In test set, the combined model also showed higher prediction performance. CT-based radiomics signature is a powerful predictor for early recurrence of IMCC. Preoperative model (constructed with homogeneity-AP and standard deviation-AP) and combined model (constructed with solitary, differentiation, energy-AP, inertia-AP, and percentile50th-PV) can improve the accuracy for pre-and postoperatively predicting the early recurrence of IMCC.

## Introduction

Intrahepatic mass-forming cholangiocarcinoma (IMCC) originates from the epithelial cell lining of intrahepatic bile ducts and accounts for approximately 10% of all primary liver cancers, which is inferior to only that of hepatocellular carcinoma (HCC)^1,2^. Though considered rare, its incidence has increased globally^3^. IMCC is a fatal malignancy, which often has a more aggressive tumor biology than HCC^4^. When possible, surgical resection is the only well-established treatment option for IMCC that offers the best possibility for disease cure^5^. Even after resection with curative intent, the prognosis of IMCC remains poor due to the high frequency of early recurrence^4,6^.

Thus, there is a need to identify high-risk early recurrence patients with IMCC so that a more aggressive surgery as well as a strict follow-up protocol can be established. Previous studies have suggested that clinicopathological variables, including preoperative carbohydrate antigen 19-9 (CA19-9) elevation, obstructive jaundice, tumor size, number of lesions, satellite lesions, lymph node metastases, perineural invasion, and macrovascular invasion were significantly associated with recurrence of IMCC patients after curative resection, but failed to elucidate predictive performance in clinical applications and some of these predictors can only be evaluated with postoperative pathological examination^4,7–10^.

Radiomics is gaining importance in personalized cancer treatment. It involves the extraction of quantitative features from digital medical images that enables mineable high-dimensional data to be applied in clinical decision support to provide improved diagnostic, prognostic, and predictive accuracy^11–16^. Science prior studies have reported the high prediction performance (AUC ≥ 0.80) of CT-based radiomics models to predict early recurrence of HCC^17,18^, we hypothesized that CT-based radiomics could improve the accuracy for predicting the early recurrence of IMCC.

Thus, the aim of this study was to investigate the ability of CT-based radiomics signature for pre-and postoperatively predicting the early recurrence of IMCC and develop radiomics-based prediction models.

## Materials and methods

The study protocol was in complies with the Declaration of Helsinki and acts in accordance to ICH GCP guidelines. Institutional review board of Nanjing Drum Tower Hospital, the affiliated hospital of Nanjing University Medical School approved this study and explicitly waived the informed consent due to its retrospective nature. It also clarified that authors had access to identifying patient information when analyzing the data.

### Patients

**Figure 1:**
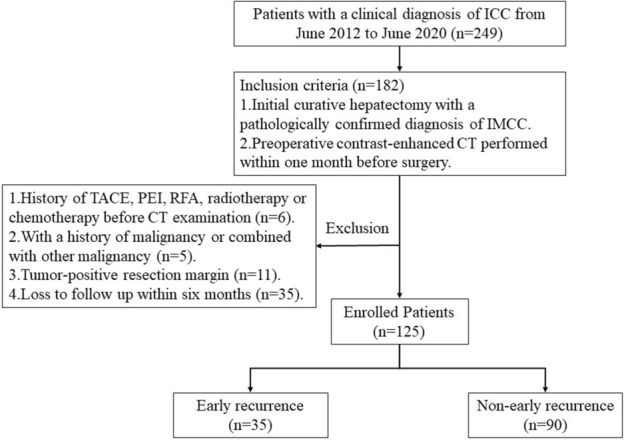
Flow chart showing inclusion and exclusion of subjects for this study. *ICC* intrahepatic cholangiocarcinoma, *IMCC* intrahepatic mass-forming cholangiocarcinoma, *TACE* transarterial chemoembolization, *PEI* percutaneous ethanol injection, *RFA* radiofrequency ablation.

From June 2012 to June 2020, a total of 249 patients with a clinical diagnosis of intrahepatic cholangiocarcinoma (ICC) were reviewed. The inclusion criteria were: (a) initial curative hepatectomy with a pathologically confirmed diagnosis of IMCC according to the 2010 World Health Organization classification; (b) preoperative contrast-enhanced CT performed within one month before surgery; (c) without any local or systematic treatment such as transcatheter arterial chemoembolization, percutaneous ethanol injection, radiofrequency ablation, radiotherapy or chemotherapy before CT examination. The exclusion criteria were: (a) with a history of malignancy or combined with other malignancy; (b) with tumor-positive resection margin; (c) those who were followed up for less than six months. The remaining 125 patients, including 67 men and 58 women, with a median age of 56 years (range 34–79) served as our study population (Fig. 1).

### CT examination

Each patient underwent plain and dynamic contrast-enhanced CT scans on a multidetector CT scanner (Lightspeed, VCT, or Discovery HD750, GE Healthcare, US). The scanning parameters are the same as the details in the previous research of our team^19^. The medium interval between CT examination and surgery was 9 days (range 4–23).

### Region-of-interest segmentation and radiomics features extraction

The region of interest (ROI) segmentation were the same as the steps described in previous study^20^. Two radiologists (Z.Y. and M.Y.F., with 7 and 5 years’ experience in abdominal radiology, respectively) manually drew along the margin of the tumor independently. The software automatically read the CT value of each pixel within the volume of interest (VOI) and generated 87 parameters as follows: (1) shape-based features describing three-dimensional size and tumor shape (n = 8); (2) the first-order statistics features describing the distribution of pixel intensity within the VOI (n = 14); (3) the texture features assessing tumor heterogeneity by analyzing the distribution and relationship of pixel or voxel gray levels in the VOI (n = 65). Radiomics features were selected by using a parametric method, the least absolute shrinkage and selection operator (LASSO) logistic regression^21–23^, after manually eliminating the features that had an absolute value less than 0.6 for the coefficients of early recurrence from the radiomics features. The other observer repeated image analysis independently as discussed 1 months later in order to assess the intra-observer reliability for the selected features.

### Basic CT imaging analysis

Basic CT imaging features of IMCCs included liver surface contour (smooth, bulging or retraction), intratumoral arteries (defined as an artery entering the tumor and remaining inside the tumor^24^) and bile duct dilatation (with or without).

Quantitative analysis was also performed. The axial CT image showing the largest slice of the tumor was selected. The first ROI was drawn manually as large as possible within the lesion area to obtain the mean CT attenuation (in Hounsfield Unit, HU), which was recorded as CT value-mean of tumor. Then, a second round/oval ROI was placed in non-tumorous liver parenchyma in the same slice as large as possible avoiding visible vessels to obtain the mean CT attenuation of liver parenchyma (in HU). The ROIs in the tumor and non-tumorous liver parenchyma were kept identical in unenhanced, arterial, portal venous and equilibrium phases. The mean values of the two radiologists were calculated as the final results. Tumor-liver ratio was calculated by CT value-mean/CT value of non-tumorous liver parenchyma in each phase.

### Surgical treatment, clinical-pathological data collection and follow-up

Sixty-one patients underwent major hepatectomy with bile duct resection and other 64 patients received partial hepatectomy without bile duct resection by two surgeons (QYD and ML, with 26 and 15 years’ experience in hepatobiliary surgery). Demographic and clinicopathological variables were collected for each patient. Solitary, size, vascular invasion, neurological and membrane invasion, lymph node metastasis and histological grade were also documented based on final pathology reports. Data on tumor stage were collected according to the AJCC seventh edition staging system^25^. After surgery, patients were followed in the outpatient clinic every 3 months for the first 2 years and every 6 months thereafter. Examinations consisted of ultrasonography and serum tumor markers, and contrast-enhanced CT, MR imaging or PET would be ordered if a recurrence or progression of the disease was suspected. Further therapeutic regimes such as radiotherapy or chemotherapy would be undertaken if disease relapse or progression was confirmed. During a median follow-up time of 22 months (mean 22.6, range 17–85), 70 patients were still alive, 46 patients died, and 9 patients were lost to follow-up. Due to the high rate of recurrence after liver resection and short median time (8–9 months) to recurrence^7,26,27^, early recurrence was defined as suspicious imaging findings or biopsy-proven tumor within 6 months.

Hence, recurrence was documented in 73 patients (73/125, 58.4%), of whom 35 relapsed within 6 months (35/73, 47.9%) and 40 relapsed after 6 months (38/73, 52.1%). In training set, 92 patients were further divided into early recurrence group (n = 24, 26.1%) and non-early recurrence group (n = 68, 73.9%). In test set, 33 patients were divided into early recurrence group (n = 11, 33.3%) and non-early recurrence group (n = 22, 66.7%).

### Statistical analysis

Categorical variables were compared by using the ×2 test or Fisher exact test. Continuous variables were compared by using the Student t test or Mann–Whitney U test, when appropriate. In training set, the selected features extracted from the VOIs and other variables that reached statistical significance (*p* value < 0.05) at the univariable analysis were considered for the multivariable logistic regression model. Receiver operating characteristics (ROC) curves were generated and the area under the curve (AUC) was used to evaluate the accuracy of preoperative model, pathological model, and combined model in predicting the early recurrence of IMCC. Then, the developed models were validated by assessing their prediction performance in test set. Comparisons between three models were performed using the Delong test in the training and test sets. Higher prediction accuracy presented with a larger AUC and a *p* value < 0.05 (two-tailed) indicated statistical significance. Decision curve analysis (DCA) of training and test sets were conducted to determine the clinical usefulness by quantifying the net benefits at different threshold probabilities in the models. Interobserver agreement of radiomics features between two radiologists were assessed with intraclass correlation coefficients (0.000–0.200, poor; 0.201–0.400, fair; 0.301–0.600, moderate; 0.601–0.800, good; 0.801–1.000, excellent). Survival curves were drawn by using the Kaplan–Meier method, and differences of survival rates were compared with the log rank, breslow, and tarone ware test. Other statistical analyses were performed with SPSS, version 22.0 (IBM, Armonk, NY, USA) and R software (R Foundation for Statistical Computing, version 3.4.1; https://www.r-project.org/).

### Ethics approval and consent to participate

Institutional review board of Nanjing Drum Tower Hospital, the affiliated hospital of Nanjing University Medical School, approved this study and the informed consent from patients was waived due to its retrospective nature.

## Results

### Patient characteristics

Early recurrence was detected in 28.0% (35/125) of IMCCs in our study. In training set, the baseline characteristics of patients are summarized in Table 1. Early recurrence group IMCCs were depicted as multiple lesions significantly more often than that in non-early recurrence group IMCCs (41.7% vs. 16.2%; *p* = 0.011). Early recurrence group IMCCs appear as larger size than non-early recurrence group IMCCs (*p* = 0.021). IMCCs in early recurrence group showed poorly to undifferentiated differentiation and membrane invasion more often than those in non-early recurrence group (*p* = 0.025 and 0.024, respectively). There were no significant differences in clinical characteristics between the early recurrence and non-early recurrence group IMCCs.

**Table 1:** Comparison of clinicopathological characteristics of patients with IMCC with early recurrence and non-early recurrence in training set.

Clinical-pathological characteristics	Total	Early recurrence (≤ 6 months) (n = 24)	Non-early recurrence (> 6 months) (n = 68)	*p Value*
Age (range, years)	58.37 ± 9.97 (34–79)	60.54 ± 12.54 (34–79)	57.57 ± 8.82 (34–77)	0.234
Gender (male/female)	49/43	12/12	37/31	0.710
Carcinoembryonic antigen (> / ≤ 5 ng/ml)	18/74	4/20	14/54	0.677
Carbohydrate antigen 19-9 (> / ≤ 37 U/ml)	45/47	11/13	34/34	0.726
Carbohydrate antigen 125 (> / ≤ 35 U/ml)	15/77	4/20	11/57	0.955
Abdominal pain (with/without)	38/54	12/12	26/42	0.314
HBV + (yes/no)	57/35	17/7	40/28	0.297
Solitary (yes/no)	71/21	14/10	57/11	0.011*
Size (range, cm)	5.82 ± 2.65(1.5–12.0)	6.85 ± 2.96(2.4–12.0)	5.44 ± 2.44(1.5–12.0)	0.021*
Differentiation				0.025*
Well to moderately differentiated	56	10	46	
Poorly to undifferentiated	36	14	22	
Vascular invasion (yes/no)	34/58	12/12	22/46	0.124
Neurological invasion (yes/no)	64/28	18/6	46/22	0.501
Membrane invasion (yes/no)	47/45	17/7	30/38	0.024*
Tumor stage (I–II/III–IV)	58/34	12/12	46/22	0.124
T stage				0.583
T1–2	69	17	52	
T3–4	23	7	16	
N stage (N0/N1)	68/24	16/8	52/16	0.347

TNM stage according to Japanese TNM stage of intrahepatic cholangiocarcinoma.

*p < 0.05.

**Figure 2:**
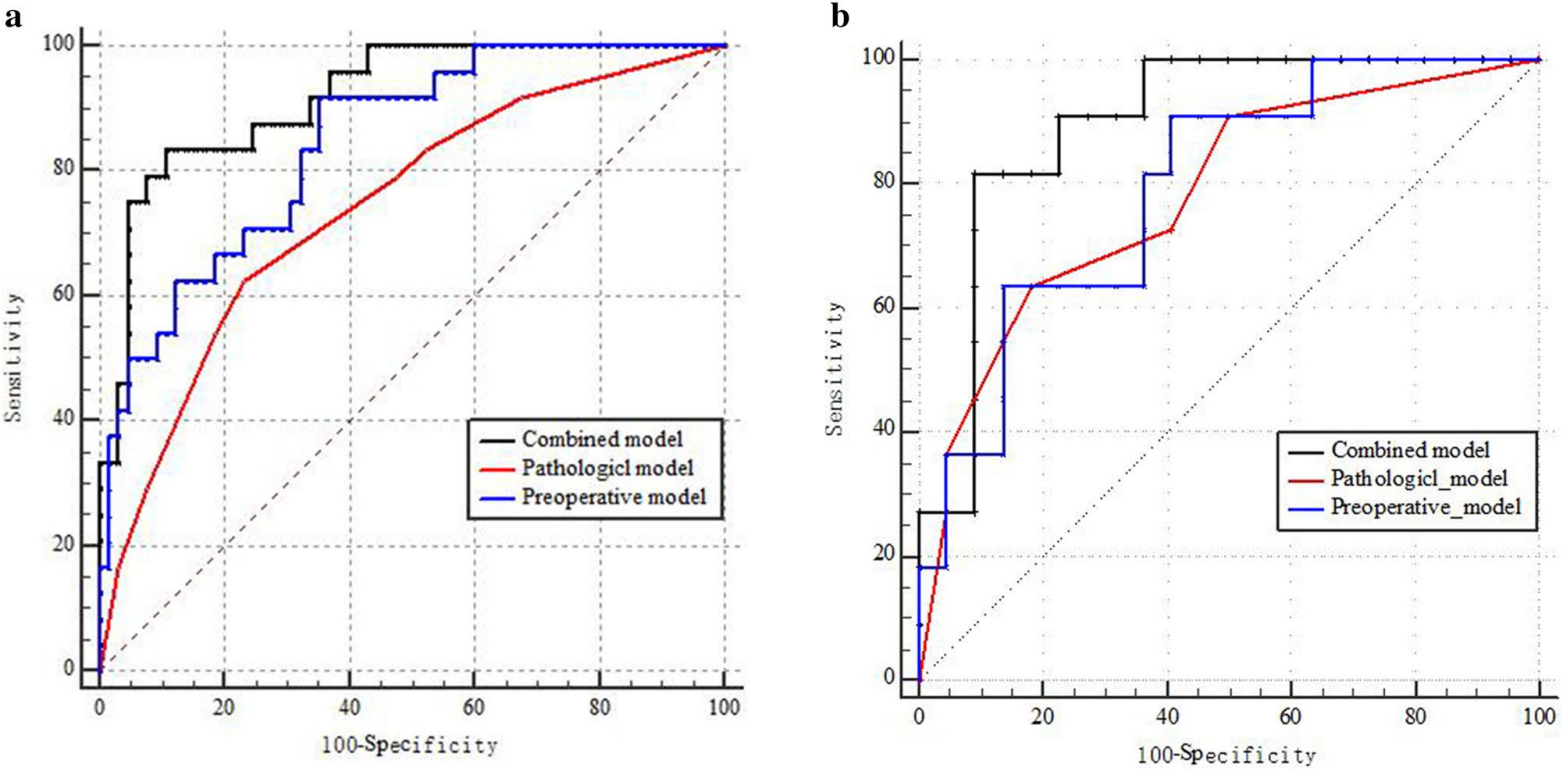
Kaplan–Meier survival curves of IMCC patients with and without early recurrence in the training set (**a**) and test set (**b**).

Overall survival in IMCC patients with early recurrence was significantly poorer than those without early recurrence both in training set (11.9 ± 2.0 vs. 64.0 ± 4.7 months, *p* < 0.001) and test set (16.8 ± 3.9 vs. 74.4 ± 6.0 months, *p* < 0.001) (Fig. 2a,b).

### Development and performance of prediction models

Based on basic CT imaging features and clinical indicators in training set (Table A.1), PV CT value-mean and EP CT value-mean were significantly associated with early recurrence. However, neither of them were identified as independent risk factors for early recurrence by multivariable analysis (*p* > 0.05).

Of 348 extracted radiomics features in plain, arterial, portal venous, and equilibrium phase CT images, 20 most stable features (15 first-order features and 5 texture features) were considered for subsequent analysis. All texture features showed excellent interobserver agreement with intraclass correlation coefficients ≥ 0.80 (Table A.2). Radiomics features in AP (homogeneity-AP and standard deviation-AP) constructed the preoperative model (AUC = 0.844; 95% CI 0.751–0.912) (Table 2), all basic CT imaging features and clinical indicators were excluded in multivariate logistic regression analysis.

**Table 2:** Logistic regression analysis for predicting early recurrence of IMCC based on basic CT imaging features, radiomics features and clinical indicators (Preoperative model).

Preoperative model	Univariate analysis		Multivariate analysis	
	*p*	Hazard ratio	*p*	Hazard ratio
Plain CT value-mean	0.356	0.968 (0.904,1.037)		
Plain tumor-liver ratio	0.927	1.002 (0.959,1.047)		
AP CT value-mean	0.894	0.997 (0.958,1.038)		
AP tumor-liver ratio	0.798	1.005 (0.969,1.041)		
PV CT value-mean	0.036*	0.975 (0.952,0.998)		
PV tumor-liver ratio	0.532	0.994 (0.976,1.012)		
EP CT value-mean	0.036*	0.973 (0.949,0.998)		
EP tumor-liver ratio	0.226	0.983 (0.955,1.011)		
Intratumoral artery	0.866	0.922 (0.358,2.374)		
Liver surface contour	0.846	1.100 (0.419,2.886)		
Bile duct dilatation	0.425	0.672 (0.254,1.783)		
Homogeneity-AP	/	/	0.030*	0.000 (0.000,0.045)
Standard deviation-AP	/	/	0.007*	1.355(1.085,1.692)
Age	0.234	1.030 (0.981,1.082)		
Gender	0.710	1.267 (0.499,3.218)		
CEA	0.677	0.771 (0.227,2.623)		
CA199	0.726	0.872 (0.342,2.221)		
CA125	0.955	0.993 (0.270,3.230)		
Abdominal pain	0.314	1.615 (0.632,4.126)		
HBV	0.297	1.700 (0.623,4.640)		

*PV* portal venous phase, *EP* equilibrium phase.

*p < 0.05, AP arterial phase.

Multivariate logistic regression analysis identified 2 independent risk factors in pathological model for predicting early recurrence of IMCC in training set, including solitary and membrane invasion (Table 3). The area under the ROC curve was considered normal (0.741; 95% CI 0.637–0.828). Multivariate logistic regression analysis based on basic CT imaging features, radiomics features, clinical indicators, and pathological characteristics identified solitary, differentiation, energy-AP, inertia-AP, and percentile50th-PV to construct combined model for predicting early recurrence of IMCC (AUC = 0.917; 95% CI 0.840–0.965) (Table 4).

**Table 3:** Logistic regression analysis for predicting early recurrence of IMCC based on pathological characteristics (Pathological model).

Pathological features	Univariate analysis		Multivariate analysis	
	*p*	Hazard ratio	*p*	Hazard ratio
Solitary	0.011*	3.701 (1.312,10.439)	0.034*	3.285 (1.092,9.886)
Size	0.021*	1.236 (1.032,1.480)		
Differentiation	0.025*	2.927 (1.124,7.625)		
Vascular invasion	0.124	2.238 (0.864,5.794)		
Neurological invasion	0.501	1.435 (0.500,4.118)		
Membrane invasion	0.024*	3.266 (1.198,8.903)	0.027*	3.309 (1.143,9.581)
Tumor staging	0.124	2.091 (0.810,5.395)		
T stage	0.583	1.338 (0.471,3.799)		
N stage	0.347	1.767 (0.634,4.920)		

*p < 0.05.

**Table 4:** Logistic regression analysis for predicting early recurrence of IMCC based on pathological characteristics, preoperative CT features and clinical indicators (Combined model).

All	Univariate analysis		Multivariate analysis	
	*p*	Hazard ratio	*p*	Hazard ratio
Solitary	0.013*	3.701 (1.312,10.439)	0.013*	10.000 (1.633,61.214)
Size	0.021*	1.236 (1.032,1.480)		
Differentiation	0.028*	2.927 (1.124,7.625)	0.020*	6.500 (1.337,31.609)
Membrane invasion	0.021*	3.266 (1.198,8.903)		
PV CT value-mean	0.036*	0.975 (0.952,0.998)		
EP CT value-mean	0.036*	0.973 (0.949,0.998)		
Energy-AP	/	/	0.015*	0.300 (0.300,0.055)
Inertia-AP	/	/	0.036*	0.720 (0.530,0.978)
Percentile50th-PV	/	/	0.044*	0.911 (0.832,0.997)

*PV* portal venous phase, *EP* equilibrium phase.

*p < 0.05.

**Figure 3:**
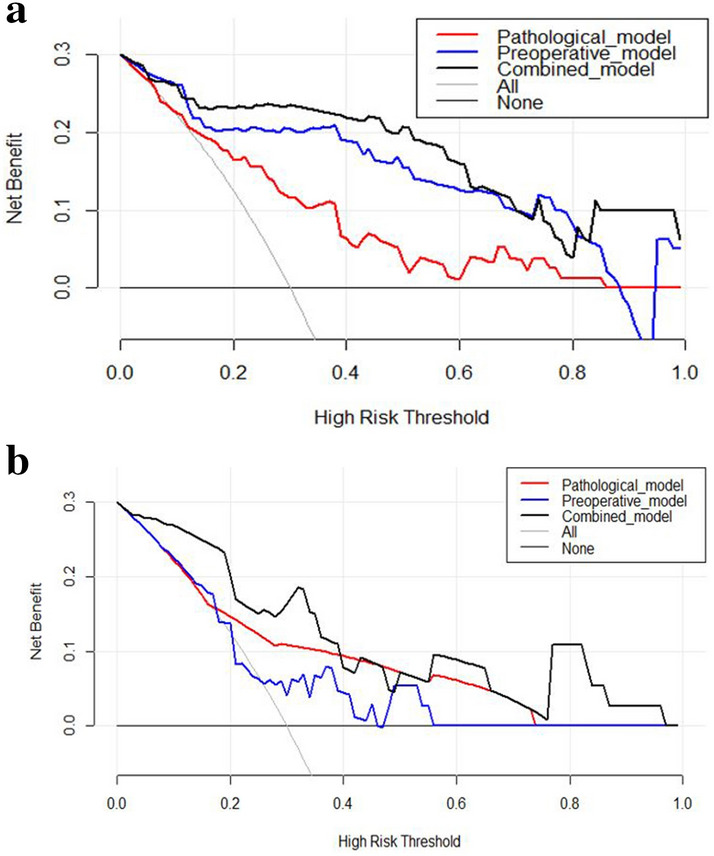
Delong non-parametric approach, AUC estimates for predicting early recurrence of IMCC were compared between different prediction models in the training set (**a**) and test set (**b**).

AUC estimates were compared between prediction models by using the Delong nonparametric approach in training and test sets (Fig. 3a,b). In training set, the AUC of the combined model was significantly higher than that of the preoperative model (*p* = 0.049) and pathological model (*p* = 0.002). In test set, the combined model yielded the excellent AUC (0.897; 95% CI 0.741, 0.975), an accuracy of 0.878, a sensitivity of 0.818, a specificity of 0.909 (Table 5).

**Table 5:** Predictive performance of the radiomics signature and the two prediction models for the discrimination of early recurrence in training set and test set.

	Variables and models	SEN	SPE	ACC	AUC (95% CI)	*p Value*		
						1 versus 2	1 versus 3	2 versus 3
Training set	1. Preoperative model	0.916	0.646	0.716	0.844 (0.751,0.912)	0.144	0.049*	0.002*
	2. Pathological model	0.625	0.767	0.730	0.741 (0.637,0.828)			
	3. Combined model	0.833	0.892	0.877	0.917 (0.840,0.965)			
Test set	1. Preoperative model	0.909	0.591	0.697	0.793 (0.617, 0.914)	0.911	0.099	0.157
	2. Pathological model	0.636	0.818	0.757	0.781 (0.603, 0.905)			
	3. Combined model	0.818	0.909	0.878	0.897 (0.741, 0.975)			

*AUC* area under the curve, *CI* confidence interval.

*p < 0.05.

### Clinical application

**Figure 4:**
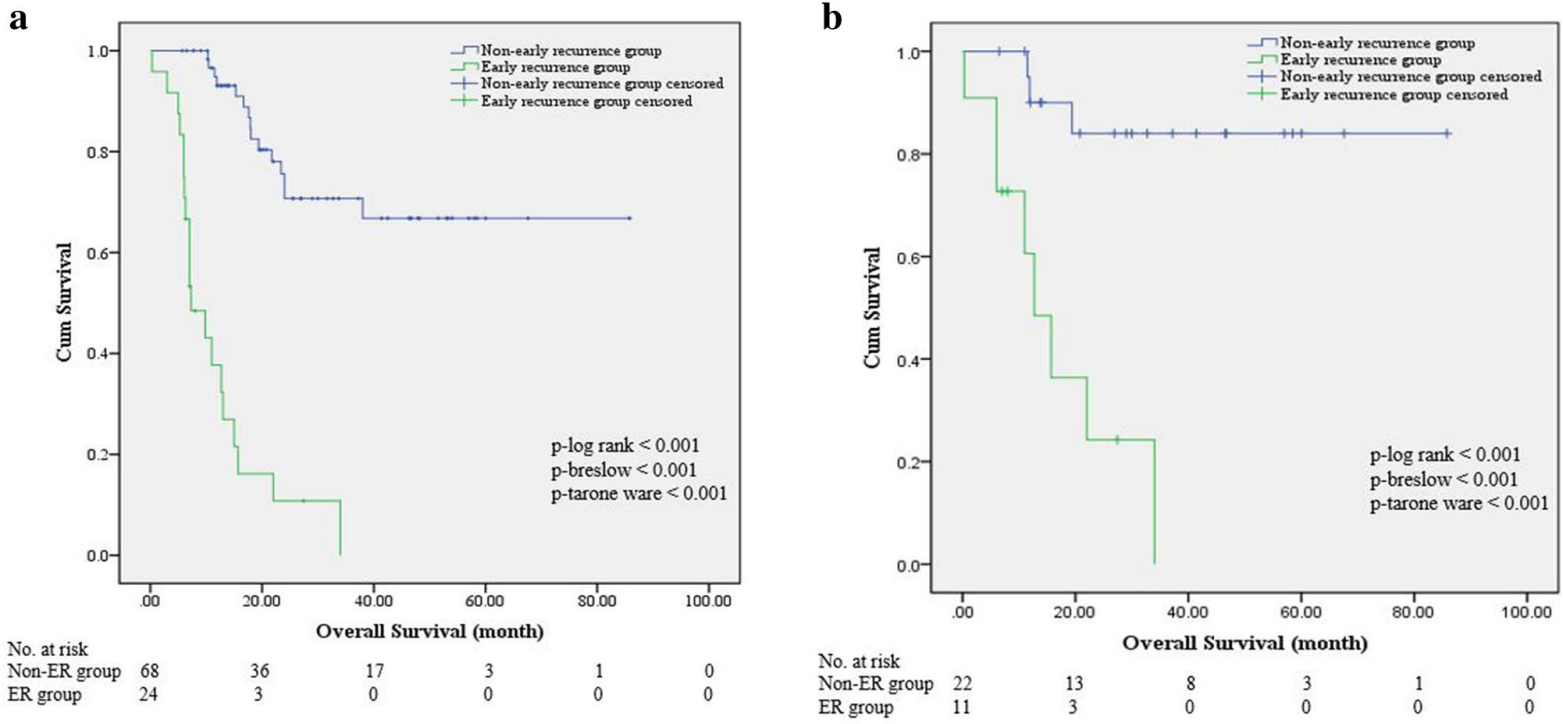
Decision Curve Analysis in the training set (**a**) and test set (**b**), decision curves of the pathological model, preoperative model and combined model.

DCA in training set and test set for the preoperative model, pathological model and combined model was performed (Fig. 4a,b). Compared with scenarios in which no prediction model would be used (i.e., treat-all or treat-none scheme), the highest curve (representing the combined model) at most of given threshold probability is the optimal decision-making strategy to maximize the net benefit compared with other two models.

## Discussion

Based on basic CT imaging features, radiomics features, clinical indicators and pathological characteristics, we developed preoperative model, pathological model, and combined model to predict the early recurrence of IMCC. Finally, the prediction performance of different models were compared.

Early recurrence (≤ 6 months) was detected in 28.0% of IMCCs in our study, which was basically consistent with previous studies^4,26,28^, and nearly half of IMCC patients had recurrence within 6 months of surgery. Due to the high rate and short median time of recurrence^7,26,27^, we suggested that predicting early recurrence of IMCC with the cutoff time of 6 months would help improve clinical prognosis. We confirmed that the prognosis of IMCC patients with early recurrence was significantly poorer than those without. Park et al.^29^ found that HCC patients with early recurrences (≤ 6 months) showed a poorer prognosis than those who did not. Zhang et al.^4^ defined 24 months as the optimal cutoff value for early and late recurrence of ICC identified with linear regression. Ohira et al.^27^ divided ICC after curative resection into early recurrence and late recurrence based on time of 12 months and found no prognostic difference between them. Determination of the optimal cutoff value for early and late recurrence of IMCC require further investigation. In addition, we found that larger tumor size, multiple lesions, poorly to undifferentiated differentiation and membrane invasion were associated with increased risk of early recurrence. Zhang et al.^4^ concluded that early recurrence occurred more commonly in ICC patients with tumors that had more aggressive biological features.

In our study, multivariate logistic regression analysis identified homogeneity-AP and standard deviation-AP to construct the preoperative mode for predicting early recurrence of IMCC (AUC = 0.844; 95% CI 0.751–0.912). However, when based on basic CT imaging features and clinical indicators, none of them were identified as independent risk factors. This result indicated that radiomics approach could generate more potentially informative and relevant metrics than basic CT imaging features and clinical indicators^11,17,18^. Early recurrence of IMCC might be related to the aggressiveness of the tumor, which could better reflect the heterogeneity inside the tumor, and radiomics can better reflect this internal heterogeneity. Indeed, two dominant features (homogeneity and standard deviation) in the preoperative mode quantified intra-tumor heterogeneity and distribution of pixel values, which were reported to be associated with histopathologic and prognosis in other solid tumors^30,31^. Accurately identifying high-risk early recurrence patients with IMCC by preoperative model could provide a basis for developing more aggressive surgery and neoadjuvant chemotherapy.

In our study, multivariate logistic regression analysis identified solitary and membrane invasion as independent risk factors in pathological model for predicting early recurrence of IMCC (AUC = 0.741), which was lower than AUC of the preoperative model (*p* = 0.144). We extracted shape-based, first-order statistics and texture features from whole tumor volume based on multiphase contrast enhanced CT imaging^32^, which completely reflect tumor microstructures and heterogeneity and might provide more valuable information reflecting the biological characteristics than several routine pathological features recorded. Prior studies have also reported the advantages of CT-based radiomics models to predict early recurrence of liver tumors. Zhou et al.^17^ developed a CT-based radiomics model that demonstrated an AUC of 0.82 in predicting the early recurrence (≤ 1 year) of HCC. Shan et al.^18^ supposed that CT-based peritumoral radiomics model can effectively predict early recurrence in HCC after curative tumor resection or ablation.

In our study, multivariate logistic regression analysis identified solitary, differentiation, energy-AP, inertia-AP and percentile50th-PV to construct combined model for predicting early recurrence of IMCC (AUC = 0.917). The high AUC in training set and test set suggested that the combined model performed well in discriminating for early recurrence and which improved the accuracy compared with either preoperative model or pathological model individually. Notably, DCA showed that the combined model adds more benefit to predicting early recurrence than the preoperative model or pathological model at most given threshold probability. For postoperative IMCC patients with high-risk early recurrence identified by combined model, it was significant to adopt more stringent follow-up protocol to detect and manage lesions early.

Our study had some limitations. First, the proposed combined model was established on the basis of data obtained from a single center. Prospective multicenter studies with considerably large datasets are needed to further validate the robustness and reproducibility of our prediction model. Second, CT images were obtained from multiple CT scanners, which might bring some potential bias. Nevertheless, a good inter-scanner agreement of CT-based radiomics method has been confirmed^33^. Furthermore, logistic regression analysis did not find significant correlation between basic CT imaging features and early recurrence of IMCCs. Combining different CT and MRI imaging characteristics to predict early recurrence requires further investigations.

## Conclusion

Our study indicates that CT-based radiomics signature is a powerful predictor for early recurrence of IMCC. Preoperative model (constructed with homogeneity-AP and standard deviation-AP) and combined model (constructed with solitary, differentiation, energy-AP, inertia-AP and percentile50th-PV) can improve the accuracy for pre-and postoperatively predicting the early recurrence of IMCC, which may help better stratify patients for surgery and enable clinicians to select optimal treatment strategies and an individualized follow-up protocol after initial surgery to improve clinical outcomes.

## Supplementary Information

Supplementary Information.

## Data Availability

The datasets during and/or analysed during the current study available from the corresponding author on reasonable request.
