# Supplementary material for: Radiomics-based model for predicting early recurrence of intrahepatic mass-forming cholangiocarcinoma after curative tumor resection
Source: Sci Rep. 2021 Sep 15;11:18347. doi: 10.1038/s41598-021-97796-1 (PMC8443588; doi:10.1038/s41598-021-97796-1)
Supplement: Supplementary file 1 — Supplementary Information. [file 41598_2021_97796_MOESM1_ESM.docx]

| Table A.1. Logistic regression analysis for predicting early recurrence of IMCC based on basic CT imaging features and clinical indicators in training set | | | | |
| --- | --- | --- | --- | --- |
| Preoperative model | Univariate Analysis | | Multivariate Analysis | |
|  | p | Hazard Ratio | p | Hazard Ratio |
| Plain CT value-mean | 0.356 | 0.968 (0.904,1.037) |  |  |
| Plain tumor-liver ratio | 0.927 | 1.002 (0.959,1.047) |  |  |
| AP CT value-mean | 0.894 | 0.997 (0.958,1.038) |  |  |
| AP tumor-liver ratio | 0.798 | 1.005 (0.969,1.041) |  |  |
| PV CT value-mean | 0.036* | 0.975 (0.952,0.998) | 0.419 | 0.982 (0.941,1.026) |
| PV tumor-liver ratio | 0.532 | 0.994 (0.976,1.012) |  |  |
| EP CT value-mean | 0.036* | 0.973 (0.949,0.998) | 0.566 | 0.986 (0.941,1.034) |
| EP tumor-liver ratio | 0.226 | 0.983 (0.955,1.011) |  |  |
| Intratumoral artery | 0.866 | 0.922 (0.358,2.374) |  |  |
| Liver surface contour | 0.846 | 1.100 (0.419,2.886) |  |  |
| Bile duct dilatation | 0.425 | 0.672 (0.254,1.783) |  |  |
| Age | 0.234 | 1.030 (0.981,1.082) |  |  |
| Gender | 0.710 | 1.267 (0.499,3.218) |  |  |
| CEA | 0.677 | 0.771 (0.227,2.623) |  |  |
| CA199 | 0.726 | 0.872 (0.342,2.221) |  |  |
| CA125 | 0.955 | 0.993 (0.270,3.230) |  |  |
| Abdominal pain | 0.314 | 1.615 (0.632,4.126) |  |  |
| HBV | 0.297 | 1.700 (0.623,4.640) |  |  |
| * *p* < 0.05  AP arterial phase, PV portal venous phase, EP equilibrium phase | | | | |

| Table A.2. Inter-observer agreement of selected texture parameters in multiphase contrast-enhanced CT imaging | | | |
| --- | --- | --- | --- |
| Feature | ICC | Feature | ICC |
| Correlation-AP | 0.870 | Percentile5th-PV | 0.994 |
| Homogeneity-AP | 0.937 | Mean-PV | 0.993 |
| Energy-AP | 0.971 | Max frequency-PV | 0.990 |
| Entropy-AP | 0.965 | Percentile10th-EP | 0.979 |
| Entropy(H) -AP | 0.949 | Percentile25th-EP | 0.966 |
| Inertia-AP | 0.933 | Percentile50th-EP | 0.969 |
| Standard deviation-AP | 0.936 | Percentile5th-EP | 0.965 |
| Percentile10th-PV | 0.990 | Percentile75th-EP | 0.888 |
| Percentile25th-PV | 0.994 | Mean-EP | 0.996 |
| Percentile50th-PV | 0.953 | Mode-EP | 0.982 |
| ICC interclass correlation coefficients. | | | |
